# Efficacy of distal perigastric lymphadenectomy for Siewert type II adenocarcinoma of the esophagogastric junction

**DOI:** 10.3389/fsurg.2025.1645297

**Published:** 2025-10-14

**Authors:** Zonglin Li, Yi Liu, Yifan Jiang, Min Song

**Affiliations:** 1 Department of General Surgery (Gastrointestinal Surgery), The Affiliated Hospital of Southwest Medical University, Luzhou, China; 2 Department of Laboratory Medicine, The Affiliated Hospital of Southwest Medical University, Luzhou, China

**Keywords:** adenocarcinoma of the esophagogastric junction, Siewert type II, distal perigastric lymph nodes, total gastrectomy, proximal gastrectomy

## Abstract

**Background:**

To investigate the metastatic rates of distal perigastric lymph nodes (DPLN), including No.3b, 4d, 5, 6 and 12a LN stations, and to evaluate the clinical significance of DPLN lymphadenectomy for patients with Siewert type II adenocarcinoma of the esophagogastric junction (AEG).

**Methods:**

From January 2014 to December 2018, 217 patients with Siewert type II AEG who underwent total gastrectomy (TG) or proximal gastrectomy (PG) were retrospectively included. Based on clinicopathological data from TG patients, the metastatic rates and the therapeutic value (TV) indexes of DPLN, along with risk factors for DPLN metastasis, were assessed. Additionally, the 5-year overall survival (OS) rates were compared between TG and PG patients.

**Results:**

The metastatic rates of No.3b, 4d, 5, 6, 12a LN stations and DPLN were 31.7%, 9.6%, 12.6%, 4.0%, 3.1% and 36.4%, whereas the 5-year TV indexes of them were 10.3, 0.0, 1.5, 0.0, 0.0 and 9.7, respectively. Tumor size (>4 cm), pT stage (pT4) and pN stage (pN3) were significant risk factors for patients with DPLN metastasis. For patients with tumor size larger than 4 cm, pT4 stage or pN3 stage, TG was associated with a better prognosis than PG, with the 5-year OS rates of 33.5% and 16.8%, respectively (χ^2^ = 4.299, *p* = 0.038).

**Conclusions:**

DPLN metastasis is a poor prognostic factor for patients with Siewert type II AEG. For high-risk patients with tumor size larger than 4 cm, cT4 stage or extensive LN metastasis identified preoperatively or intraoperatively, it is recommended to perform TG with expanded lymphadenectomy, including resection of DPLN.

## Introduction

In recent years, the incidence of adenocarcinoma of the esophagogastric junction (AEG) has been greatly increasing worldwide (1–3). Compared to typical gastric cancers, AEGs possess distinct anatomical characteristics and exhibit more aggressive behavior (4). Therefore, as a kind of independent disease, AEGs have increasingly gained the attention of gastrointestinal and thoracic surgeons (5–7). The Siewert classification is widely used to categorize AEGs into three types: tumors with an epicenter of 1–5 cm above the esophagogastric junction (EGJ) are Siewert type I AEGs, 1 cm above to 2 cm below EGJ are Siewert type II AEGs, and 2–5 cm below the EGJ are Siewert type III AEGs (8). Currently, surgery remains the most important treatment for resectable AEGs and there is a consensus that Siewert type I and III AEGs should adhere to the surgical therapeutic principles of lower esophageal cancers (transthoracic subtotal esophagectomy with proximal gastrectomy) and proximal gastric cancers (transabdominal total gastrectomy), respectively (5). However, the optimal surgical approach for Siewert type II AEGs remains controversial, particularly regarding the choice between total gastrectomy (TG) and proximal gastrectomy (PG), because the necessity of dissecting distal perigastric lymph nodes (DPLN), including No.3b, 4d, 5, 6 and 12a LN stations, remains under debate (9).

According to the Japanese gastric cancer treatment guidelines 2021 (6th edition), radical lymphadenectomy in TG demands complete removel of No.1, 2, 3a, 3b, 4sa, 4sb, 4d, 5, 6, 7, 8a, 9, 11p, 11d and 12a LN stations, whereas PG only demands removel of No.1, 2, 3a, 4sa, 4sb, 7, 8a, 9, 11p and 11d LN stations (10). So DPLN lymphadenectomy is only included in radical TG but not in PG. Also, in the Japanese gastric cancer treatment guidelines 2021 (6th edition), AEGs refer to Siewert types II AEGs and both TG and PG can be used as conventional surgical methods (10). Nevertheless, omitting DPLN lymphadenectomy in PG for patients with bigger-sized or later-staged tumors maybe compromise oncological safety. Given this concern, we designed this study to investigate the metastatic rates of DPLN and assess the clinical significance of DPLN lymphadenectomy for patients with Siewert type II AEG.

## Materials and methods

### Patients

**Figure 1:**
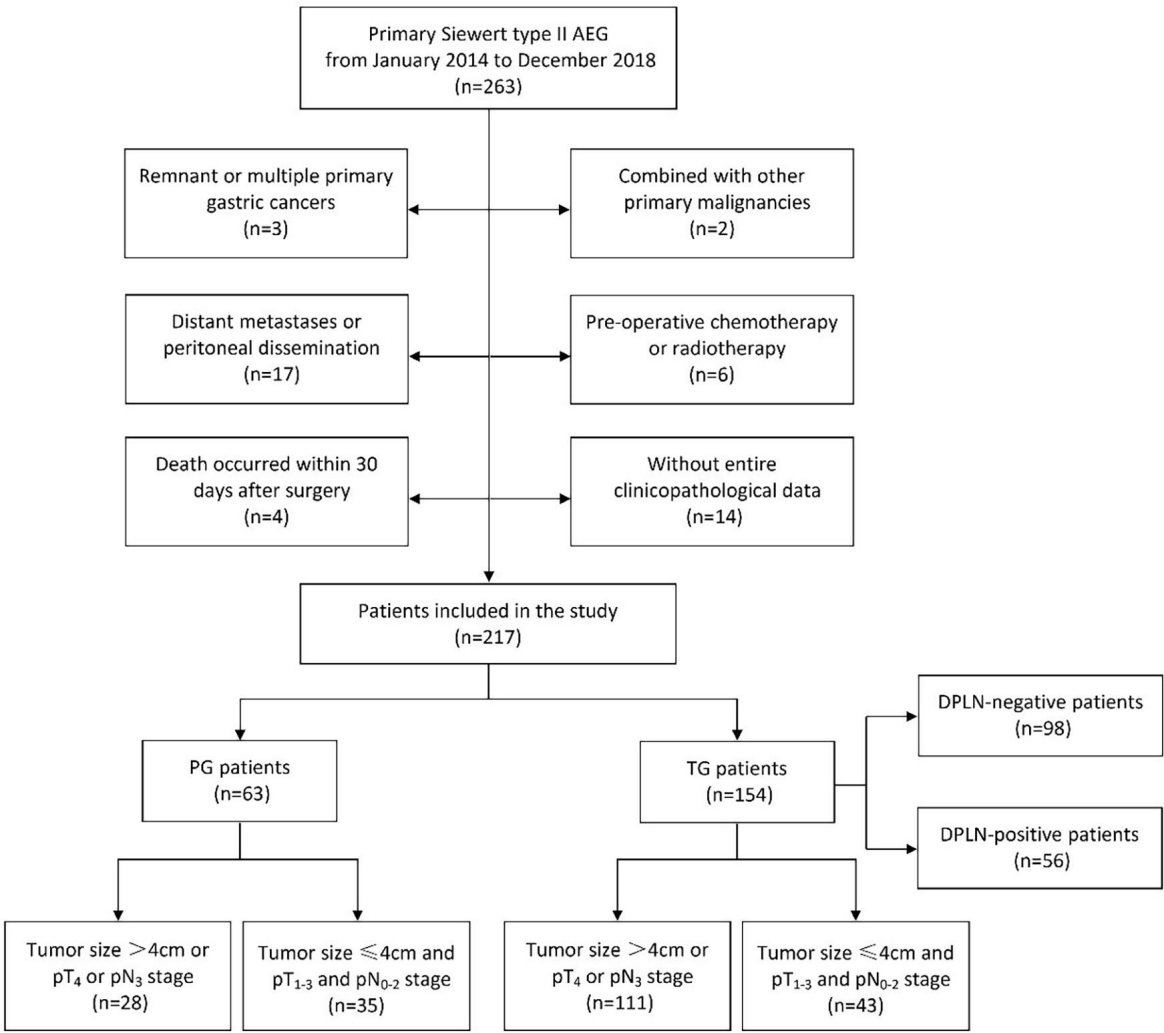
The flowchart of patients with Siewert type II AEG included in this study. AEG, adenocarcinoma of esophagogastric junction; TG, total gastrectomy; PG, proximal gastrectomy; DPLN, distal perigastric lymph nodes.

From January 2014 to December 2018, 263 patients with Siewert type II AEG who underwent transabdominal open radical gastrectomy at The Affiliated Hospital of Southwest Medical University were retrospectively included. The inclusion criteria were as follows: (1) patients had a clear postoperative pathologic diagnosis of primary Siewert type II AEG; (2) patients received transabdominal open radical gastrectomy with standard lymphadenectomy; and (3) patients in whom the number of harvested LNs were more than 16 nodes. The exclusion criteria were as follows: (1) remnant or multiple primary gastric cancers; (2) combined with other primary malignancies; (3) distant metastases or peritoneal dissemination; (4) pre-operative chemotherapy or radiotherapy; (5) death occurred within 30 days after surgery; and (6) without entire clinicopathological data. After applying these criteria, 217 patients (154 patients with TG and 63 patients with PG) were included in this study. This retrospective study strictly complied with the Declaration of Helsinki and was approved by the Clinical Ethics Committee of The Affiliated Hospital of Southwest Medical University. Informed consent was obtained from all the participants. All the data used in the study was appropriately anonymized before analysis. A flowchart of the patients included in the study was shown in Figure 1.

### Surgical management and follow-up

All the patients were diagnosed by preoperative gastroscopy and pathological biopsy, and preoperative computed tomography were performed to evaluate the tumors' clinical stage. Preoperative doctor-patient communication was carried out and the differences of surgical extent as well as postoperative quality of life between TG and PG were explained for all patients. Whether performing TG or PG were mainly based on the patients' preference and the surgeon's suggestion, which was mainly referred the T stage of tumors, DPLN status and the size of residual stomach. When both TG and PG were selectable, the patients were responsible for determining the surgical procedure and signing the consent form. Transabdominal open radical gastrectomy was performed for all the patients according to Japanese gastric cancer treatment guidelines 2014 (ver. 4) (11) or 2018 (5th edition) (12). Generally, for TG the extent of lymphadenectomy included the resection of No.1, 2, 3, 4sa, 4sb, 4d, 5, 6, 7, 8a, 9, 10, 11p, 11d and 12a LN stations, while for PG the extent of lymphadenectomy included the resection of No.1, 2, 3, 4sa, 4sb, 7, 8a, 9 and 11p LN stations, a small subset of patients underwent resection of No.19, 20, 110 and 111 LN stations. All the operations were performed by an experienced gastric cancer surgeon. The oral safety margin at the esophagus was usually 2 cm under the naked eye. In case of doubt, intraoperative frozen section examination was performed to ensure the esophageal margin was negative. The perigastric LNs were checked out from the excised specimens as much as possible after operation. The diagnosis of Siewert type II AEG was confirmed by postoperative specimen anatomy and pathologic diagnosis. Tumor sizes were determined by pathological measurement from the resected specimen and divided into two groups for analysis (≤4 cm vs. >4 cm). Postoperative chemotherapy (generally oxaliplatin with capecitabine) was recommended for patients with advanced tumor and 185 (85.3%) patients completed postoperative chemotherapy at last.

All the patients were periodically followed up by outpatient visits or telephone interviews after surgery. The follow-up was performed every 3 months during the first 2 postoperative years, every 6 months during the subsequent 3 years and every year thereafter until death. Survival time was calculated from surgery to the last contact (June 2024) or death. Among the 217 patients, 206 (94.9%) cases had complete follow-up.

### Evaluation of the therapeutic value index for lymph nodes

Based on clinicopathological data from TG patients, the metastatic rates and the therapeutic value (TV) indexes for DPLN were calculated, and risk factors for DPLN metastasis were analyzed. The TV index, proposed by Sasako et al. (13), estimates the benefit of LN resection by multiplying the metastatic rate of each LN station by the 5-year (or 2-year in this study) overall survival (OS) rate of patients with positive LNs at that station. The metastatic rate and the OS rate of patients with positive LNs were calculated independently for each LN station, regardless of metastasis to other LN stations and without any reference to the overall pathological nodal stage (13–15).

### Survival analysis

For TG patients, the 5-year OS rates between DPLN-positive and DPLN-negative groups were compared and the prognostic factors of patients with Siewert type II AEG were assessed. What's more, the postoperative survival difference between TG and PG groups for patients with Siewert type II AEG were also assessed.

### Statistical analysis

All statistics analyses were performed using the SPSS software (version 26.0). Chi-square or Fisher's exact test was performed to analyze unordered categorical variables, while Mann–Whitney *U*-test was applied to evaluate ordinal categorical variables. Logistic regression model was used to analyze the risk factors of DPLN metastasis. The OS rates were calculated using the Kaplan–Meier method and differences between groups were compared by the Log-rank test. Univariate and multivariate survival analyses were performed by Cox's proportional hazard regression model with conditional backward stepwise. A two-sided *p*-value <0.05 was considered statistically significant.

## Results

### Metastatic rates and TV indexes of DPLN for patients with Siewert type II AEG

As illustrated in Table 1, based on clinicopathological data from TG patients, the metastatic rate, the 2-year and 5-year OS rates as well as the 2-year and 5-year TV indexes for each LN station were calculated. It was clear that the metastatic rates and the TV indexes of No.1, 2 and 3a LN stations were much higher than that of other LN stations.

**Table 1:** Metastatic rate and TV index of each LN station for TG patients with Siewert type II AEG.

LN station	LN-positive patients (*n*)	Total patients (*n*)	Metastatic rate (%)	2-year OS rate (%)	2-year TV index	5-year OS rate (%)	5-year TV index
No.1	58	143	40.6	67.2	27.3	37.9	15.4
No.2	60	139	43.2	63.3	27.3	33.3	14.4
No.3a	62	142	43.7	77.4	33.8	38.7	16.9
No.3b	46	145	31.7	69.6	22.1	32.6	10.3
No.4sa	49	128	38.3	61.2	23.4	28.6	10.9
No.4sb	36	130	27.7	55.6	15.4	22.2	6.2
No.4d	13	136	9.6	61.5	5.9	0.0	0.0
No.5	17	135	12.6	47.1	5.9	11.8	1.5
No.6	5	126	4.0	0.0	0.0	0.0	0.0
No.7	50	148	33.8	66.0	22.3	32.0	10.8
No.8a	16	144	11.1	62.5	6.9	12.5	1.4
No.9	38	134	28.4	68.4	19.4	28.9	8.2
No.10	4	97	4.1	50.0	2.1	0.0	0.0
No.11p	18	137	13.1	50.0	6.6	16.7	2.2
No.11d	13	128	10.2	46.2	4.7	7.7	0.8
No.12a	3	98	3.1	0.0	0.0	0.0	0.0
No.19	4	47	8.5	75.0	6.4	50.0	4.3
No.20	5	54	9.3	80.0	7.4	40.0	3.7
No.110	2	43	4.7	50.0	2.3	0.0	0.0
No.111	2	37	5.4	50.0	2.7	0.0	0.0
DPLN	56	154	36.4	69.6	25.3	26.8	9.7

TV, therapeutic value; LN, lymph node; TG, total gastrectomy; AEG, adenocarcinoma of esophagogastric junction; OS, overall survival; DPLN, distal perigastric lymph nodes.

In terms of LN metastatic rate, the most common metastatic sites were No.1, 2 and 3a LN stations, (rates >40%), while the least common were No.6, 10, 12a, 110, and 111 LN stations (rates ≤6%). Although No.6 (4.0%) and No.12a (3.1%) LN stations showed relatively lower metastatic rates for patients with Siewert type II AEG, individually. The metastatic rate of No.3b (31.7%) LN station was approximate to No.4sb (27.7%), No.7 (33.8%) and No.9 (28.4%) LN stations, and the metastatic rate of No.4d (9.6%) and No.5 (12.6%) LN stations were approximate to No.8a (11.1%), No.11p (13.1%), No.11d (10.2%), No.19 (8.5%) and No.20 (9.3%) LN stations. Moreover, the metastatic rate of DPLN which was composed of No.3b, 4d, 5, 6 and 12a LN stations could yield a higher metastatic rate of 36.4%.

The TV indexes were calculated for evaluating the value of LN resection. In terms of the 2-year TV indexes, although they were 0.0 for No.6 and No.12a LN stations, the 2-year TV indexes of No.3b (22.1), No.4d (5.9) and No.5 (5.9) LN stations were approximate to most other LN stations. In terms of the 5-year TV indexes, the TV indexes of No.1, 2 and 3a LN stations were highest (all >14.0), whereas the TV indexes of No.4d, 6, 10, 12a, 110 and 111 LN stations were 0.0; the TV index of No.3b (10.3) LN station was approximate to No.4sa (10.9), No.4sb (6.2), No.7 (10.8) and No.9 (8.2) LN stations; and the TV index of No.5 (1.5) LN station was approximate to No.8a (1.4), No.11p (2.2), and No.11d (0.8) LN stations; the overall TV index of DPLN which was composed of No.3b, 4d, 5, 6 and 12a LN stations was 9.7.

### Risk factors of DPLN metastasis for patients with Siewert type II AEG

As demonstrated in Table 2, the risk factors of DPLN metastasis for patients with Siewert type II AEG were analyzed by Logistic regression model. In the univariate analyses, the involved variables significantly consisted of the following clinicopathologic factors: tumor size (>4 cm, OR = 3.947, *p* < 0.001), tumor differentiation (poorly and undifferentiated, OR = 1.977, *p* = 0.046), pT stage (pT4, OR = 13.444, *p* < 0.001) and pN stage (pN3, OR = 18.578, *p* < 0.001). Finally, multivariate analysis by Logistic regression suggested that tumor size (>4 cm, OR = 6.028, *p* = 0.015), pT stage (pT4, OR = 17.076, *p* < 0.001) and pN stage (pN3, OR = 23.131, *p* < 0.001) were independent risk factors of DPLN metastasis for patients with Siewert type II AEG.

**Table 2:** Logistic regression analysis of the risk factors for DPLN metastasis in TG patients with Siewert type II AEG.

Factors	Univariate analysis		Multivariate analysis	
	OR (95% CI)	*P*-value	OR (95% CI)	*P*-value
Gender (Male/Female)	1.333 (0.690–2.577)	0.392	–	–
Age (≤60 years/>60 years)	0.881 (0.453–1.716)	0.710	–	–
BMI (≤24/>24)	0.990 (0.513–1.910)	0.976	–	–
Tumor size (≤4 cm/>4 cm)	3.947 (1.945–8.013)	<0.001	6.028 (1.418–25.627)	0.015
Macroscopic type (Bormmann I–II/III–IV)	0.788 (0.406–1.530)	0.482	–	–
Tumor differentiation (Well, moderately/Poorly, undifferentiated)	1.977 (1.013–3.857)	0.046	0.727 (0.166–3.183)	0.672
CEA (≤6 ng/ml/>6 ng/ml)	1.350 (0.686–2.658)	0.385	–	–
pT stage (T1–T3/T4)	13.444 (6.040–29.926)	<0.001	17.076 (5.474–53.271)	<0.001
pN stage (N0-2/N3)	18.578 (7.849–43.971)	<0.001	23.131 (7.162–74.708)	<0.001
Vascular invasion (No/Yes)	1.828 (0.926–3.607)	0.082	–	–
Nerve invasion (No/Yes)	1.467 (0.738–2.916)	0.275	–	–

DPLN, distal perigastric lymph nodes; TG, total gastrectomy; AEG, adenocarcinoma of esophagogastric junction; OR, odds ratio; CI, confidence interval; BMI, body mass index; CEA, carcinoembryonic antigen; –, not enter the regression model.

### Survival analysis of DPLN-positive and DLPLN-negative patients

**Figure 2:**
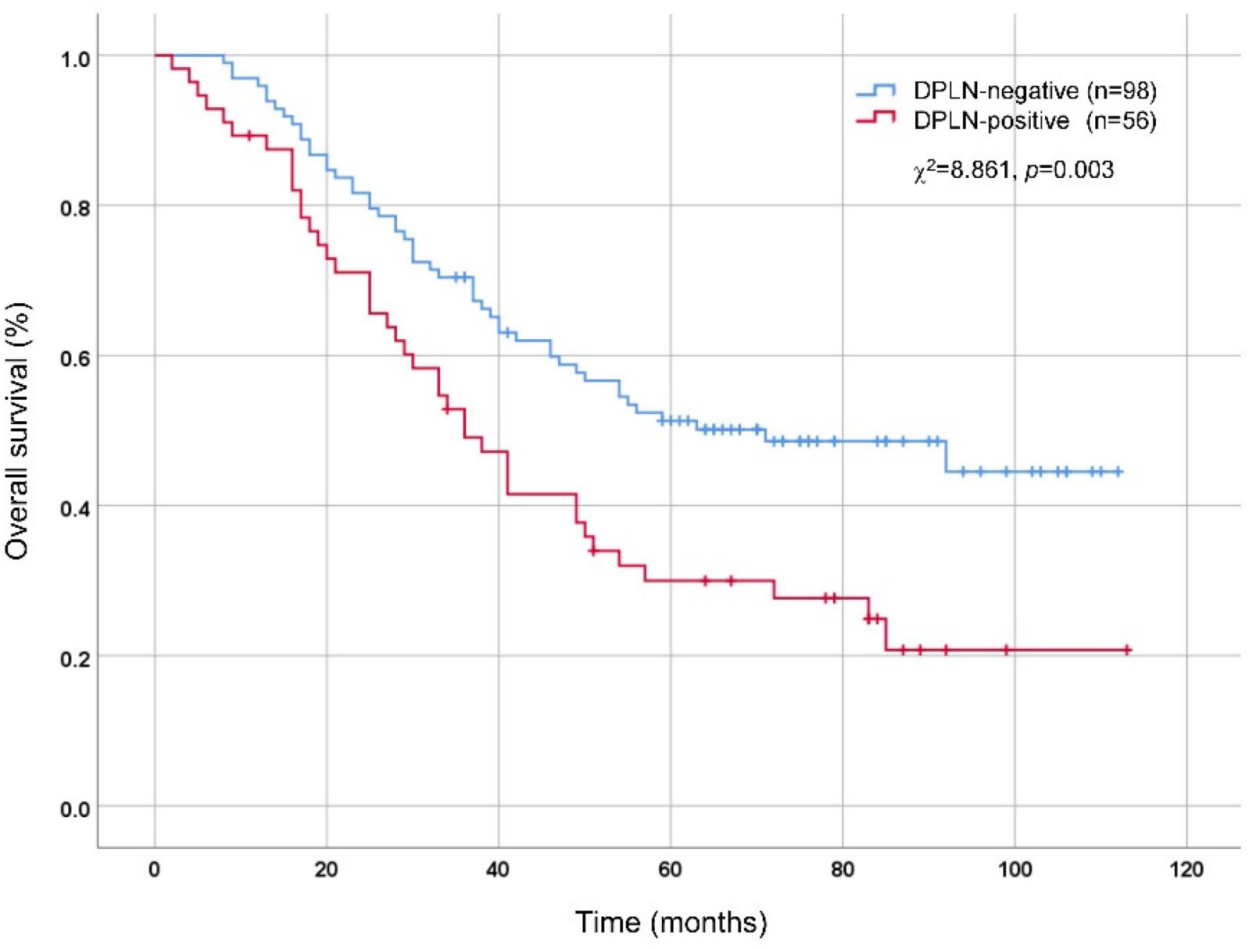
Comparison of overall survival between DPLN-positive and DPLN-negative patients by Kaplan-Meier method and Log-rank test. DPLN, distal perigastric lymph nodes.

As illustrated in Table 3, the clinicopathological factors were comparable between the DPLN-positive and DLPLN-negative patients (*p* > 0.05), except for the high risk factors of DPLN metastasis, including tumor size (*p* < 0.001), tumor differentiation (*p* = 0.044), pT stage (*p* < 0.001) and pN stage (*p* < 0.001). According to OS analysis by Kaplan–Meier method and Log-rank test, DPLN-positive patients showed worse prognosis than DPLN-negative patients. The 5-year OS rates for DPLN-positive and DPLN-negative patients were 30.0% and 51.3%, respectively (χ^2^ = 8.861, *p* = 0.003), as illustrated in Figure 2.

**Table 3:** Comparison of clinicopathological features by Chi-square test between DPLN(+) and DPLN(−) groups for TG patients with Siewert type II AEG.

Variable	DPLN(+) group *n* (%)	DPLN(−) group *n* (%)	*P*-value
Total	56 (36.4)	98 (63.6)	
Gender			
Male	28 (50.0)	56 (57.1)	0.392
Female	28 (50.0)	42 (42.9)	
Age (year)			
≤60	24 (42.9)	39 (39.8)	0.710
>60	32 (57.1)	59 (60.2)	
BMI			
≤24	27 (48.2)	47 (48.0)	0.976
>24	29 (51.8)	51 (52.0)	
Tumor size (cm)			
≤4	16 (28.6)	60 (61.2)	<0.001
>4	40 (71.4)	38 (38.8)	
Macroscopic type			
Bormmann I/II	33 (58.9)	52 (53.1)	0.481
Bormmann III/IV	23 (41.1)	46 (46.9)	
Tumor differentiation			
Well/moderately	22 (39.3)	55 (56.1)	0.044
Poorly/undifferentiated	34 (60.7)	43 (43.9)	
CEA (ng/ml)			
≤6	20 (35.7)	42 (42.9)	0.385
>6	36 (64.3)	56 (57.1)	
pT stage			
T1–3	12 (0.0)	77 (6.1)	<0.001
T4	44 (78.5)	21 (21.4)	
pN stage			
N0-2	18 (0.0)	88 (12.3)	<0.001
N3	38 (67.8)	10 (10.2)	
Vascular invasion			
No	31 (48.2)	68 (69.4)	0.080
Yes	25 (51.8)	30 (30.6)	
Nerve invasion			
No	34 (60.7)	68 (69.4)	0.274
Yes	22 (39.3)	30 (30.6)	
Postoperative chemotherapy			
No	11 (19.6)	12 (12.2)	0.215
Yes	45 (80.4)	86 (87.8)	

DPLN, distal perigastric lymph nodes; TG, total gastrectomy; AEG, adenocarcinoma of esophagogastric junction; BMI, body mass index; CEA, carcinoembryonic antigen.

Moreover, the Cox proportional hazards model was used to evaluate the prognostic significance of DPLN for patients with Siewert type II AEG. As shown in Table 4, the Cox proportional hazards model for multivariate analysis revealed that DPLN (DPLN-negative vs. DPLN-positive, HR = 1.963, *p* = 0.045) combined with tumor size (≤4 cm vs. >4 cm, HR = 2.640, *p* = 0.002), pT stage (pT1-3 vs. pT4, HR = 2.161, *p* = 0.006) and pN stage (pN0-2 vs. pN3, HR = 2.538, *p* = 0.001) were independent prognostic factors for patients with Siewert type II AEG. However, clinicopathological factors, such as age, gender, body mass index (BMI), macroscopic type, tumor differentiation, carcinoma embryonic antigen (CEA), vascular invasion, nerve invasion and postoperative chemotherapy were not significant independent prognostic factors according to multivariate Cox regression analysis (*p* >0.05), even though some of them were closely associated with the OS in univariate analysis.

**Table 4:** Univariate and multivariate Cox regression risk analysis of overall survival for TG patients with Siewert type II AEG.

Factors	Univariate analysis		Multivariate analysis	
	HR (95% CI)	*P-*value	HR (95% CI)	*P*-value
Gender (Male/Female)	1.466 (0.971–2.213)	0.069	–	–
Age (≤60 years/>60 years)	0.821 (0.542–1.242)	0.351	–	–
BMI (≤24/>24)	1.219 (0.806–1.846)	0.348	–	–
Tumor size (≤4 cm/>4 cm)	2.332 (1.512–3.596)	<0.001	2.640 (1.412–4.937)	0.002
Macroscopic type (Bormmann I–II/III–IV)	0.895 (0.590–1.358)	0.603	–	–
Tumor differentiation (Well, moderately/Poorly, undifferentiated)	1.529 (1.009–2.316)	0.045	0.860 (0.475–1.555)	0.617
CEA (≤6 ng/ml/>6 ng/ml)	1.075 (0.704–1.640)	0.739	–	–
pT stage (T1–T3/T4)	2.021 (1.337–3.057)	0.001	2.161 (1.252–3.731)	0.006
pN stage (N0-2/N3)	2.305 (1.513–3.509)	<0.001	2.538 (1.474–4.370)	0.001
Vascular invasion (No/Yes)	0.850 (0.550–1.312)	0.462	–	–
Nerve invasion (No/Yes)	0.803 (0.514–1.255)	0.336	–	–
Postoperative Chemotherapy (No/Yes)	0.549 (0.329–0.914)	0.021	0.667 (0.392–1.134)	0.135
DPLN (Negative/Positive)	1.850 (1.223–2.800)	0.004	1.963 (1.016–3.792)	0.045

TG, total gastrectomy; AEG, adenocarcinoma of esophagogastric junction; HR, hazard ratio; CI, confidence interval; BMI, body mass index; CEA, carcinoembryonic antigen; DPLN, distal perigastric lymph nodes; –, not enter the regression model.

### Comparison of postoperative survival between TG and PG patients

**Figure 3:**
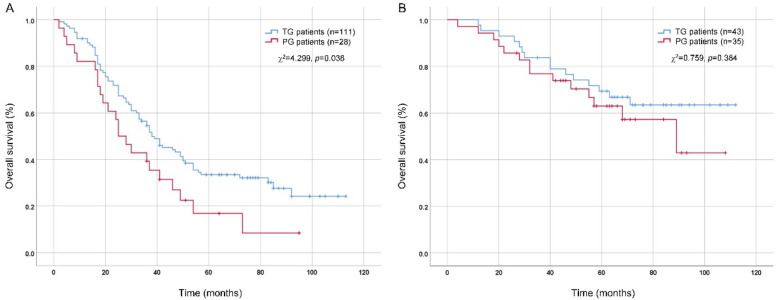
Comparison of overall survival between TG and PG patients by Kaplan-Meier method and log-rank test. **(A)** patients with tumor size >4 cm or pT4 stage or pN3 stage; **(B)** patients with tumor size ≤4 cm and pT1-3 stage and pN0-2 stage. TG, total gastrectomy; PG, proximal gastrectomy.

As multivariate analysis by Logistic regression suggested that tumor size (>4 cm), pT stage (pT4) and pN stage (pN3) were independent risk factors of DPLN metastasis for patients with Siewert type II AEG, tumor size (≤4 cm vs. >4 cm), pT stage (pT1-3 vs. pT4) and pN stage (pN0-2 vs. pN3) were set as the cut-off values for subgroup analysis. As shown in Table 5, the clinicopathological factors were comparable between TG and PG groups (*p* > 0.05), except for pN stage (*p* = 0.034) in the high-risk subgroup (tumor size >4 cm, or pT4 stage, or pN3 stage). As illustrated in Figure 3A, for patients with tumor size larger than 4 cm or pT4 stage or pN3 stage, TG patients were illustrated to have a better prognosis than PG patients, with the 5-year OS rates 33.5% and 16.8%, respectively (χ^2^ = 4.299, *p* = 0.038). However, as illustrated in Figure 3B, for patients with tumor size less than 4 cm and pT1-3 stage and pN0-2 stage, although there is a tendency for TG to have a better prognosis, the 5-year OS rates between TG and PG patients were not significant (*p* > 0.05).

**Table 5:** Comparison of clinicopathological features by Chi-square test (when counts less than 5 in the contingency table, fisher's exact test was used) between TG and PG groups for patients with Siewert type II AEG.

Variable	Tumor size >4 cm or pT4 stage or pN3 stage			Tumor size ≤4 cm and pT1-3 stage and pN0-2 stage		
	TG patients *n* (%)	PG patients *n* (%)	*P*-value	TG patients *n* (%)	PG patients *n* (%)	*P*-value
Total	111 (79.9)	28 (20.1)		43 (55.1)	35 (44.9)	
Gender						
Male	58 (52.3)	15 (53.6)	0.901	26 (60.5)	21 (60.0)	0.967
Female	53 (47.7)	13 (46.4)		17 (39.5)	14 (40.0)	
Age (year)						
≤60	45 (40.5)	11 (39.3)	0.904	18 (41.9)	14 (40.0)	0.868
>60	66 (59.5)	17 (60.7)		25 (58.1)	21 (60.0)	
BMI						
≤24	50 (45.0)	12 (42.9)	0.835	24 (55.8)	16 (45.7)	0.375
>24	61 (55.0)	16 (57.1)		19 (44.2)	19 (54.3)	
Tumor size (cm)						
≤4	33 (29.7)	10 (35.7)	0.540	43 (100.0)	35 (100.0)	–
>4	78 (70.3)	18 (64.3)		0 (0.0)	0 (0.0)	
Macroscopic type						
Bormmann I/II	56 (50.5)	17 (60.7)	0.331	29 (67.4)	24 (68.6)	0.915
Bormmann III/IV	55 (49.5)	11 (39.3)		14 (32.6)	11 (31.4)	
Tumor differentiation						
Well/moderately	41 (36.9)	8 (28.6)	0.408	36 (83.7)	27 (77.1)	0.463
Poorly/undifferentiated	70 (63.1)	20 (71.4)		7 (16.3)	8 (22.9)	
CEA (ng/ml)						
≤6	39 (35.1)	10 (35.7)	0.954	23 (53.5)	15 (42.9)	0.350
>6	72 (64.9)	18 (64.3)		20 (46.5)	20 (57.1)	
pT stage						
T1–3	46 (41.4)	15 (53.6)	0.248	43 (100.0)	35 (100.0)	–
T4	65 (58.6)	13 (46.4)		0 (0.0)	0 (0.0)	
pN stage						
N0-2	63 (56.8)	22 (78.6)	0.034	43 (100.0)	35 (100.0)	–
N3	48 (43.2)	6 (21.4)		0 (0.0)	0 (0.0)	
Vascular invasion						
No	69 (62.2)	15 (53.6)	0.406	30 (69.8)	22 (62.9)	0.520
Yes	42 (37.8)	13 (46.4)		13 (30.2)	13 (37.1)	
Nerve invasion						
No	71 (64.0)	16 (57.1)	0.505	31 (72.1)	24 (68.6)	0.734
Yes	40 (36.0)	12 (42.9)		12 (27.9)	11 (31.4)	
Postoperative chemotherapy						
No	18 (16.2)	5 (17.9)	0.835	5 (11.6)	4 (11.4)	0.978
Yes	93 (83.8)	23 (82.1)		38 (88.4)	31 (88.6)	

TG, total gastrectomy; PG, proximal gastrectomy; AEG, adenocarcinoma of esophagogastric junction; BMI, body mass index; CEA, carcinoembryonic antigen.

## Discussion

AEGs are biologically aggressive and most diagnosed at advanced stages, so patients with AEG usually have worse prognosis than patients with distal gastric cancer (4, 16). Currently, surgery remains the mainstay treatment for resectable AEGs and a worldwide consensus exists that transthoracic subtotal esophagectomy with PG and transabdominal TG are the standard surgical procedures for Siewert type I and III AEGs, respectively (5, 17, 18). However, due to the unique anatomic location for Siewert type II AEGs, there still are many controversies involving surgical therapy, one of which is the choice of gastrectomy pattern, TG or PG, because whether performing lymphadenectomy of DPLN, including No.3b, 4d, 5, 6 and 12a LN stations, remains under debate (9, 19). In this study, we investigated the metastatic rates and TV indexes of DPLN, and assess the clinical significance of DPLN lymphadenectomy for patients with Siewert type II AEG. The results and conclusions may provide some evidence for the choice of gastrectomy pattern and the extent of lymphadenectomy.

The status of the perigastric LNs is a key prognostic factor and radical lymphadenectomy can significantly improve the long-term OS for AEG patients (14). Moreover, whether or not the resected LNs have metastasis, sufficient resection of perigastric LNs is significant for the accurate staging of tumors and the decision of subsequent treatment (20, 21). According to the Japanese gastric cancer treatment guidelines 2021 (6th edition), TG and PG are both selectable for Siewert type II AEGs and the guidelines indicates that removal of No. 3b LN station is not mandatory when performing PG (10). We were concerned about the oncological safety of this surgical procedure for patients with bigger-sized or later-staged tumors, so we designed this study to assess the clinical significance of DPLN lymphadenectomy for patients with Siewert type II AEG.

Consistent with previous studies (22, 23), our study also showed higher metastatic rates and TV indexes for No.1, 2, 3a, 4sa, 4sb, 7 and 9 LN stations, indicating that the resection of these LN stations was beneficial. It is worth noting that the metastatic rate of No.3b (31.7%) LN station was approximate to these LN stations and the metastatic rate of No.4d (9.6%) and No.5 (12.6%) LN stations were approximate to No.8a, 11p, 11d, 19 and 20 LN stations. Moreover, the metastatic rate of DPLN which was composed of No.3b, 4d, 5, 6 and 12a LN stations could yield a higher metastatic rate of 36.4%. However, some retrospective studies have suggested that DPLN metastasis is rare, questioning the need for TG (24, 25). Recently, a prospective nationwide multicentre study in Japan, aiming to determine the optimal extent of lymphadenectomy for Siewert type II AEG based on the metastatic rates of perigastric LNs: category-1 (>10%, strongly recommended for resection), category-2 (5%–10%, weakly recommended), and category-3 (<5%, not recommended) (26). In that study, the metastatic rates of distal perigastric LNs (No.4d, 5 and 6 LN stations) were all less than 5%, but the metastatic rate of at least one of No.4d, 5, or 6 LN stations reached 10.7% when tumor size was bigger than 6 cm and TG was recommended to ensure oncological safety (26). However, the metastatic rates of DPLN in our study were relative higher. The reason for this difference may be the fact that there were more patients with bigger-sized and later-staged tumors in China. And if following this principle, No.3b, 4d and 5 LN stations must be removed for Siewert type II AEG based on our data. Although the 5-year TV indexes of No.4d, 6 and 12a LN stations were 0.0, it does not mean that resection of these LNs is of no value. Because there indeed exists metastatisis in these LNs, including No.4d (9.6%), No.6 (4.0%) and No.12a (3.1%), and the TV indexes maybe influenced by the insufficient sample size or too few events. Overall, DPLN lymphadenectomy appears valuable.

Given the considerable metastatic rates of DPLN, the risk factors of DPLN metastasis were identified by Logistic regression analysis. As shown in Table 2, tumor size (>4 cm), pT stage (pT4) and pN stage (pN3) were independent risk factors of DPLN metastasis for patients with Siewert type II AEG. In addition, we analyzed the prognosis of patients with Siewert type II AEG. According to the OS analysis by Kaplan–Meier method, we found that the prognosis of DPLN-positive patients was worse than DPLN-negative patients, which suggested DPLN metastasis was a poor prognostic factor for patients with Siewert II AEG. Wang et al. (27) analyzed the metastasis of parapyloric LNs (No.5 and 6 LNs) and they maintained that parapyloric LN metastasis was a significant prognostic factor for Siewert type II AEG. In our study, DPLN status together with tumor size, pT stage and pN stage were independent prognostic factors for patients with Siewert type II AEG based on Cox proportional hazards model analysis. These results also suggested that DPLN lymphadenectomy may be profitable for patients with Siewert type II AEG.

At last, postoperative survival difference between TG and PG patients were compared. As illustrated in Figure 3A, for patients with tumor size larger than 4 cm or pT4 stage or pN3 stage, although there were more pN3 stage patients in the TG group, TG patients were illustrated to have a better prognosis than PG patients (*p* = 0.038). The reason for this result may be attributed to more complete lymphadenectomy for TG. The Japanese gastric cancer treatment guidelines 2021 (6th edition) pointed out that TG and PG are both selectable for Siewert type II AEGs (10). Mine et al. (28) pointed out that PG can be accepted when the gastric invasion length was less than 3 cm and TG should be selected when the gastric invasion length was more than 5 cm. Lin et al. (29) recommended that TG with the resection of parapyloric LNs (No.5 and 6 LNs) should be selected for Siewert type II AEG when tumor diameter exceeded 4 cm. Our results suggest that TG with DPLN lymphadenectomy, particularly the resection of No.3b, 4d and 5 LN stations, is valuable for patients with Siewert type II AEG, and this therapeutic principle is especially suitable for patients with tumor size larger than 4 cm, pT4 stage or pN3 stage. It is worth noting that PG is an acceptable surgical option for patients with tumor sizes less than 4 cm and pT1-3 stage and pN0-2 stage, and TG may be associated with significant complications, including nutritional deficiencies and dumping syndrome. Just as Hölscher A et al. emphasized: the surgical approach to Siewert type II AEG should be individualized, because there is no “one size fits all” option, and criteria for individualization are epidemiological, functional, oncologic and surgical items (30).

Our study has limitations. Firstly, the retrospective nature of our single-centre study and small sample sizes in subgroup analyses might limit the efficacy of its results. Secondly, whether performing TG or PG were mainly based on the patients' preference and the surgeon's decision, which may introduces selection bias and have an impact on the results. Therefore, large-scale prospective multicentre studies are still needed for this issue to validate these findings. Despite the limitations, this study indicates that DPLN metastasis is a poor prognostic factor for patients with Siewert type II AEG. Although TG and PG are both selectable for patients with Siewert type II AEG, for high-risk patients with tumor size larger than 4 cm, cT4 stage or extensive LN metastasis during preoperative or intraoperative evaluation, it is recommended to perform TG with expanded lymphadenectomy, including resection of DPLN.

## Data Availability

The raw data supporting the conclusions of this article will be made available by the authors, without undue reservation.
